# Supplementary material for: Predicting Maternal and Infant Tetrahydrocannabinol Exposure in Lactating Cannabis Users: A Physiologically Based Pharmacokinetic Modeling Approach
Source: Pharmaceutics. 2023 Oct 14;15(10):2467. doi: 10.3390/pharmaceutics15102467 (PMC10610403; doi:10.3390/pharmaceutics15102467)
Supplement: Supplementary file 1 [file pharmaceutics-15-02467-s001.zip › pharmaceutics-2627036-supplementary.pdf]

Predicting Maternal and Infant Tetrahydrocannabinol Exposure in Lactating Cannabis Users: A Physiologically Based Pharmacokinetic Modeling Approach

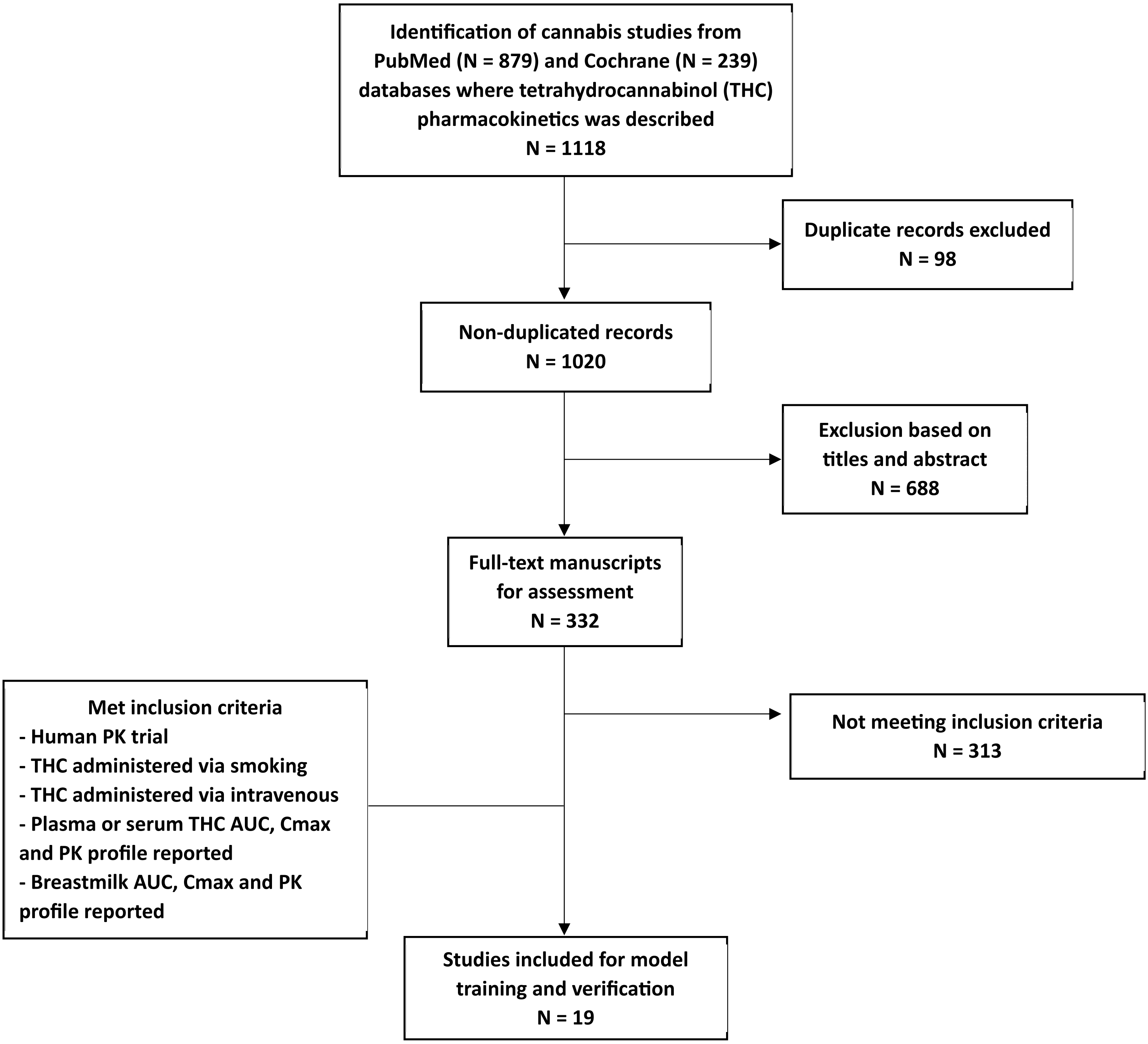

Figure S1: Flow chart of the literature review for training and verifying PBPK model predictions.
